# Spontaneous Rupture of the Renal Pelvis Secondary to Upper Tract Urothelial Carcinoma

**DOI:** 10.7759/cureus.100598

**Published:** 2026-01-02

**Authors:** Nikolay A Halachev, Stamen Andreev, Alexander Stoychev

**Affiliations:** 1 Urology, Medical Institute of the Ministry of Interior, Sofia, BGR

**Keywords:** computed tomography, renal pelvis, spontaneous rupture, urohematoma, urothelial carcinoma

## Abstract

Spontaneous rupture of the renal pelvis is a rare case of urologic emergency. In almost all instances, it occurs as a result of obstruction of the urinary tract, leading to increased pelvic pressure, most often caused by a calculus. We present a case of a 71-year-old male with flank and abdominal pain. Rupture of the renal pelvis with urohematoma was suspected, but the etiology was unknown. Imaging, retrograde ureteropyelograhy, and flexible ureterorenoscopy couldn't find the underlying cause for the suspected renal pelvis rupture, and a subsequent explorative surgery was needed in order to establish a diagnosis. Treatment included radical nephroureterctomy with bladder cuff excision. Cystoscopy and PET/CT were performed as a follow-up. No evidence of disease recurrence was found.

## Introduction

Spontaneous rupture of the renal pelvis is a rare case of urologic emergency, which is most often caused by an obstructing ureteric calculus leading to increased intrapelvic pressure [1]. Underlying causes can be divided into two groups - internal and external to the urogenital tract. The first one includes calculi, infections, iatrogenic, and tumors [1-6], and the second - trauma, malignancies, retroperitoneal fibrosis, lymph nodes, and aberrant vessels [7-12], causing an external compression leading to hydronephrosis and subsequent rupture. In the literature, however, idiopathic rupture has also been reported [13]. Our aim is to report a case of spontaneous pelvic rupture due to an obstructing urothelial carcinoma located in the pyeloureteral junction. 

## Case presentation

A 71-year-old male was admitted to the gastroenterology department due to elevated liver enzymes, poor general condition, and abdominal pain. The patient stated that the abdominal pain is located in the left upper and lower quadrants with maximum intensity in the left lumbar region. These symptoms occurred approximately 48 hours prior to admission. The patient denied any history of trauma or smoking and does not recall recent weight loss. Comorbidities include heart failure (New York Heart Association, NYHA class 2), hypertension, and mild aortic stenosis. Due to the pain in the lumbar region, a consultation with a urologist was made. 

Physical examination revealed tenderness and exacerbation of pain on palpation in the said region. The patient was also febrile, with a temperature up to 38ᵒC. Some of the more important findings in the clinical laboratory are shown in Table 1.

**Table 1:** The patient's laboratory findings *No specific bacteria was isolated from the urine or blood culture CRP - C-reactive protein; BUN - blood urea nitrogen

Blood test	Value	Normal range
Hemoglobin	92 g/L	135-180 g/L
Leukocytes	12.5x10⁹	3.5-10.5x10⁹
CRP	327 mg/L	0-5 mg/L
Iron	4.1 μmol/L	11.6-31.3 μmol/L
Iron binding capacity	38.3 μmol/L	50.4-90.7 μmol/L
Creatinine	87 μmol/L	63-111 μmol/L
BUN	5.6 mmol/L	2.8-8.3 mmol/L
Bacteria in urine*	67 μmol/L	0-33 μmol/L

**Figure 1:**
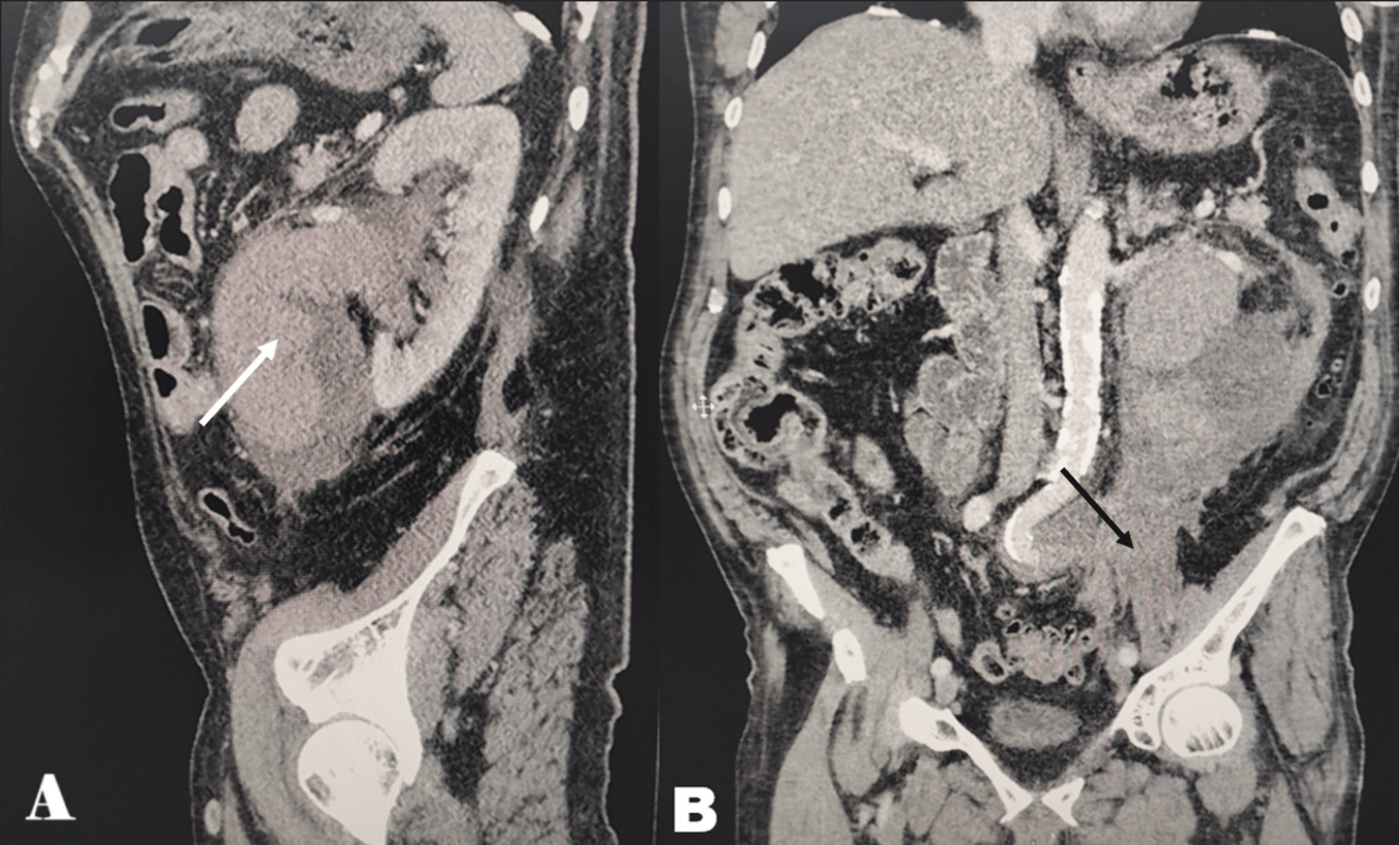
CT scan of the pelvis A: The white arrow shows hyperdense content mixed with fluid density. B: The lesion can be traced to the pelvis as pointed by the black arrow.

Rupture of the kidney pelvis was suspected, and a CT scan with contrast medium was requested (Figure 1). It showed medial and caudal to the left kidney a massive non-homogeneous lesion suggestive of urohematoma measuring 10x6x15 cm. The said lesion did not enhance after administering contrast medium; only the kidney appeared to enhance its density. Due to the presence of the suspected urohematoma, any tumor formations couldn't be distinguished. 

**Figure 2:**
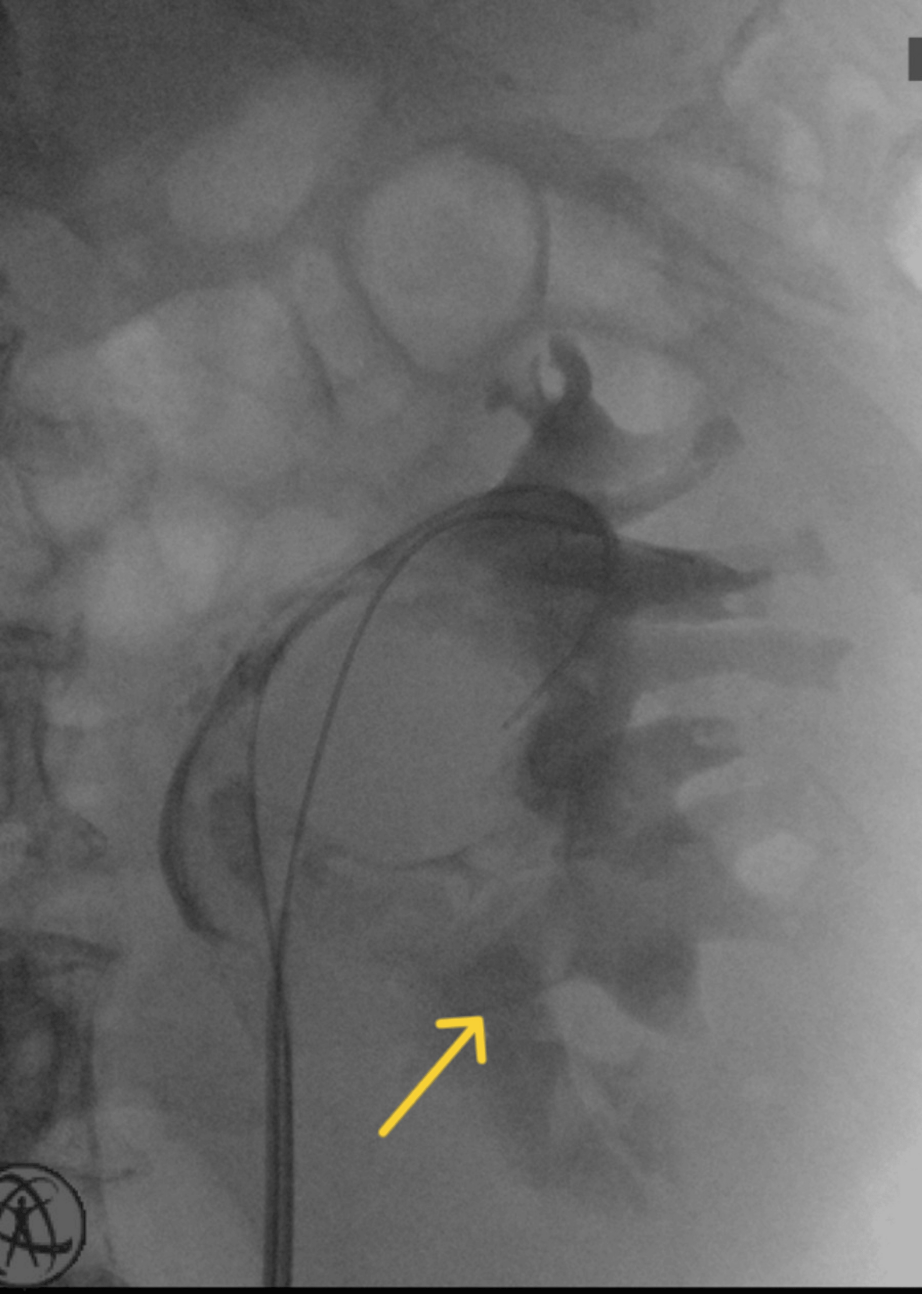
Retrograde ureteropyelography Tumor formation wasn't visualized; extravasation of the contrast medium was seen, as shown by the yellow arrow.

With these findings, highly suspicious of urinoma or perirenal hematoma, and the worsening overall condition of the patient, it was decided to proceed surgically. Retrograde ureteropyelography was conducted in order to find a cause. Extravasation of the contrast dye at the level of the kidney pelvis was seen (Figure 2). Filling defect, however, was not visualized due to the dilution of contrast medium in the already extravasated fluids. Subsequent retrograde flexible ureterorenoscopy couldn't establish an etiology for the pelvic rupture either because of the hematoma obstructing the view.

**Figure 3:**
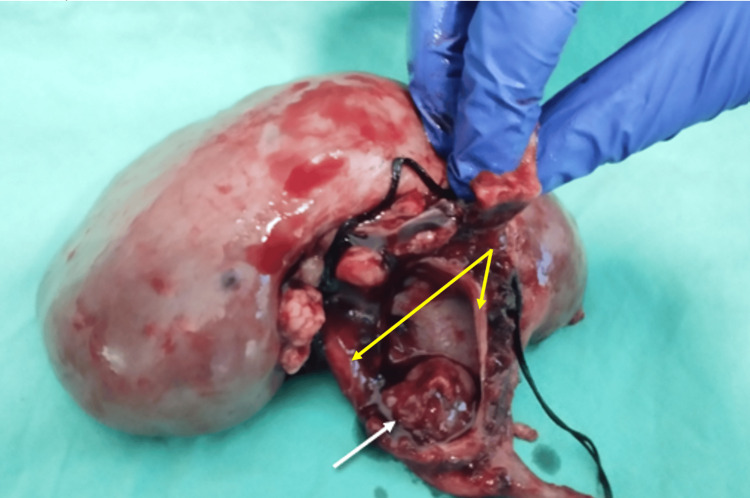
Post-nephrectomy kidney with the ruptured pelvis The white arrow shows the obstructing tumor. The yellow arrows show the edges of the ruptured pelvic wall (site of rupture).

The patient underwent an open nephroureterectomy with bladder cuff excision. During the operation, the obstructing tumor located at the pelviureteric junction was clearly visualized. Proximal to the tumor on the medial upper wall of the renal pelvis, a rupture (Figure 3) was seen as well as a surrounding perirenal urohematoma, confirming the diagnosis. Pathologic evaluation revealed G2/LG (World Health Organization/International Society of Urological Pathology, WHO/ISUP) muscle-invasive papillary urothelial carcinoma of the pelvis without lymphovascular invasion; lymph nodes were negative for metastatic disease and negative surgical margins - pT2N0M0. 

Serial follow-up three months post-surgery using cystoscopy and urine cytology, as well as a PET/CT at six months, showed no evidence of disease recurrence.

## Discussion

Spontaneous renal pelvis rupture is a rare entity that is underreported in the literature [2]. In about 80% of the cases, the cause is obstruction due to impacted stones. There are a few cases worldwide regarding spontaneous rupture of the renal pelvis due to obstruction by a tumor. Patients typically present with back pain, dysuria, and microscopic hematuria, but sometimes they can be asymptomatic. If there is suspicion for such a diagnosis, an imaging modality like a CT scan is preferred [14].

Upper tract urothelial carcinoma (UTUC) is a relatively infrequent malignancy with an incidence of 5-10% of all urothelial tumors. It is more common in male patients between 70-90 years, with >50% of them being current smokers. UTUC arises from the urothelial lining of the renal pelvis and ureter and is characterized by distinct molecular, genetic, and environmental features compared to bladder urothelial carcinoma. Tumor growth narrows or occludes the urinary tract lumen, impeding urine outflow, and as the pressure exceeds the tensile strength of the renal pelvic wall, rupture occurs. Consequently, external compression from other types of tumors, such as rectal cancer and metastatic disease, can also cause such ruptures [7,8].

Hematuria is the most frequent symptom. In addition, there is about a 29% chance of recurrence in the bladder after treatment for UTUC, which means it is mandatory to perform cystoscopic evaluation at the time of diagnosis and as follow-up [15].

Diagnostic workup of UTUC includes CT urography, which has the highest diagnostic accuracy for detecting and staging [16]. The lesions appear to be mildly hyperdense in the unenhanced phase of the CT, in contrast to the more hyperdense blood clots or stones. Due to their poor blood supply, UTUCs have little accumulation of contrast in the arterial phase. In the excretory phase, they appear as filling defects [1]. MR urogram can also be useful in the diagnostic workup.

There is a lack of evidence on how to properly treat patients with upper urinary tract ruptures [17]. Most of the cases described in the literature suggest treatment modalities ranging from conservative ones with antibiotic administration up to surgical ones like placement of a ureteral stent or a nephrostomy tube [5,14,17]. Most authors favor non-surgical treatment. Morgan et al. propose that surgical approaches are suitable for patients with signs of infection, kidney failure, or other risk factors [17].

Tumor seeding following renal pelvis rupture caused by an obstructing tumor is a concerning possible complication - viable tumor cells may implant and proliferate outside the urinary tract, potentially leading to local recurrence, peritoneal carcinomatosis, or distant metastasis as seen with transitional cell carcinoma of the bladder after perforation [18]. Tumor seeding along the percutaneous access route for treatment of UTUC has also been reported in the literature [19,20].

## Conclusions

Spontaneous rupture of the renal pelvis is a rare condition usually secondary to obstructing ureteric calculi. In a few cases, however, the underlying cause may be a urothelial tumor as seen in this case. Symptoms are not specific, ranging from mild flank pain and hematuria to an acute abdomen. It is suspected clinically and usually confirmed with appropriate imaging modalities. Early diagnosis is important in order to prevent further complications.
